# *Retraction*: Adyel *et al.* Health Risk Assessment of Pesticide Residues via Dietary Intake of Market Vegetables from Dhaka, Bangladesh. *Foods* 2013, *2*, 64–75

**DOI:** 10.3390/foods2030350

**Published:** 2013-07-31

**Authors:** Md. Shakhaoat Hossain, Md. Alamgir Hossain, Md. Abdur Rahman, Md. Mainul Islam, Md. Atiqur Rahman, Tanveer Mehedi Adyel

**Affiliations:** 1 Department of Environmental Sciences, Jahangirnagar University, Dhaka 1342, Bangladesh; 2 Department of Applied Nutrition and Food Technology, Islamic University, Kushtia 7703, Bangladesh; E-Mails: alamgirhossain8585@gmail.com (M.A.H.); arahman@bijoy.net (M.A.R.); 3 Department of Public Health and Informatics, Jahangirnagar University, Dhaka 1342, Bangladesh; E-Mail: mrahman5488@gmail.com; 4 Department of Chemistry, University of Dhaka, Dhaka 1000, Bangladesh; E-Mail: mmislam778@gmail.com; 5 School of Environmental Systems Engineering, University of Western Australia, 35 Stirling Highway, Crawley, WA 6009, Australia; E-Mail: 21193926@student.uwa.edu.au; 6 Department of Environmental Science, Z. H. Sikder University of Science & Technology, Bhedergonj, Shariatpur 8024, Bangladesh

The following article: doi: 10.3390/foods2010064, available online at: http://www.mdpi.com/2304-8158/2/1/64 [1], has been retracted by the authors because of some major errors in the broad field of identifying pesticide residue and its concentration. During random cross-check, retention time of pesticides by HPLC did not match with the standard values. As a result, the concentrations of all detected pesticides, maximum residue limits (MRLs), and health risk assessments were altered. These errors render the article [1] incorrect. All authors have confirmed that the reported results were produced using quite inappropriate procedures. As first author, I take full responsibility for the retraction and any other errors in its contents, and would like to offer my apologies on behalf of my co-authors to the readership of *Foods* for any inconvenience caused by this retraction.
